# Mucinous intestinal adenocarcinoma arising from mature cystic teratoma of the ovary treated with FOLFOX-6 - a case report

**DOI:** 10.3332/ecancer.2024.1709

**Published:** 2024-06-04

**Authors:** Enrique Bedoya-Ismodes, Karem Portugal, Sandy Carmona-Lozano, Mirna Dominguez-Gonzales, Jaime Torres, Nestor Juarez-Herrera

**Affiliations:** 1 Hospital Santa Rosa, Lima 15084, Perú; 2 Facultad de Medicina Humana, Universidad de San Martín de Porres, Lima, Perú; a https://orcid.org/0000-0002-6792-2371; b https://orcid.org/0000-0002-4851-8664; c https://orcid.org/0000-0002-8954-3727; d https://orcid.org/0000-0002-4895-6824; e https://orcid.org/0000-0002-7414-5785

**Keywords:** mature cystic teratoma, malignant transformation, mucinous intestinal adenocarcinoma, FOLFOX 6, colorectal origin

## Abstract

The malignant transformation of mature cystic teratomas during pregnancy is a rare occurrence in medical literature. In this case report, we present a remarkable instance of mucinous intestinal adenocarcinoma arising from a mature ovarian cystic teratoma diagnosed during pregnancy. This is among the few reports detailing the effective use of the FOLFOX 6 chemotherapy regimen for treating this type of intestinal cancer. Furthermore, we emphasize the immunohistochemical results that confirm the colorectal histological origin.

## Introduction

Dermoid cysts, also known as mature cystic teratomas, are among the most diagnosed ovarian neoplasms and primarily affect women during their reproductive years. These cysts have the potential to originate from any one of the three primary germ layers [1].

While most teratomas are benign tumours, they pose a notable risk of malignancy. Malignant transformation within mature ovarian cystic teratomas is rare, occurring in fewer than 2% of cases [2]. Squamous cell carcinoma represents the most common form of malignancy, accounting for about 75% of such transformations, while the progression into mucinous intestinal adenocarcinoma is particularly rare, with a reported incidence of less than 6.8% [3, 4].

This transformation may manifest with clinical symptoms, yet it might also be an incidental finding in an asymptomatic patient during pregnancy or, as described in this case, during a cesarean section [1]. This report details a rare instance of malignant conversion to mucinous intestinal adenocarcinoma within a mature ovarian cystic teratoma that remained asymptomatic. The case was diagnosed and managed by the oncology department at Hospital Santa Rosa in Lima, Peru.

## Case report

The patient, a 30-year-old woman from Venezuela, underwent an emergency cesarean section due to preeclampsia. Her medical history includes untreated hypertension, mild bronchial asthma and marijuana use. Her father was diagnosed with bladder cancer at the age of 50.

During the first-trimester ultrasound, a right ovary measuring 36 × 37 mm was identified, showing a follicle and a 26 mm mass, while the left ovary was obscured by an 80 × 50 mm complex cyst, predominantly solid (80%), indicative of a mature teratoma. Additionally, elevated levels of tumour markers were detected (Figure 1).

She underwent an emergency segmental cesarean section at 35 weeks of gestation due to severe preeclampsia. Intraoperatively, the uterus exhibited friable tissue and pinpoint erythematous lesions on the posterior surface, with ligaments adhering to the posterior surface, along with the involvement of the omentum and intestinal loops. Pinpoint lesions were also present on the ovaries. Additionally, there was 2,000 cc of citrine-yellow gelatinous fluid, alongside a tumour originating from the ovary, and a live single fetus.

Following the cesarean section, evaluation by the gynecologic oncology service recommended an exploratory laparotomy, with the possibility of cytoreduction.

Post-surgery contrast-enhanced abdominal tomography revealed retroperitoneal paraaortic and mesenteric lymph nodes measuring less than 15 × 11 mm. Additionally, a multiseptated mass measuring 190 × 85 × 125 mm was identified, with a solid component measuring 62 × 44 mm. Pelvic magnetic resonance imaging with contrast displayed a complex cystic lesion characterised by thick and irregular septa, a hemorrhagic component, and solid areas. This made measurement of the left ovary challenging, with free fluid evident in the pelvic cavity.

The laparotomy yielded suboptimal cytoreduction, and a Blake drain was inserted. The surgical findings were as follows: no hepatic metastasis, 5,600 cc of mucinous citrine fluid was drained, and a 15 × 12 cm complex, solid, multilobulated mass was observed in the left ovary. This mass was flattened and adhered to the small intestine and rectum without infiltration. A mass was observed attached to the uterus at the cervical level, seemingly infiltrating it along its entire length but not involving the bladder. Additionally, on the cecum, a flattened mass originating from the cecal appendix was noted, extending into both the right and left paracolic areas. This mass displayed necrotic regions containing mucin, causing architectural distortion and apparent infiltration throughout its entirety.

**Figure 2.**
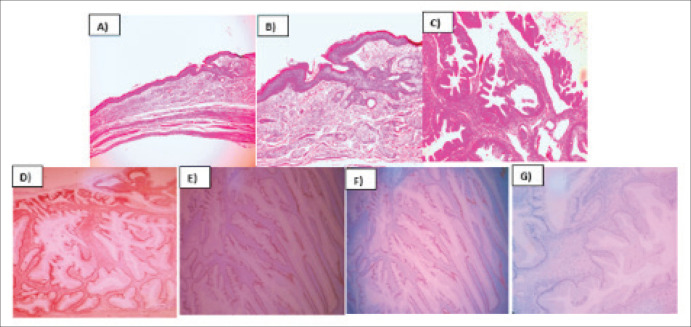
Mucinous intestinal adenocarcinoma arising from mature cystic teratoma of ovary. (a): H&E 10X, Mature cystic teratoma component of the ovary. (b): H&E 20X, Mature cystic teratoma component of the ovary. (c): H&E 40X, Malignant transformation of ovary´s cystic teratoma to well differentiated mucinous adenocarcinoma. (d): CK20, 40X, Membrane inmunoreactivity in mucinous adenocarcinoma. (e): SATB2, 40X, Nuclear inmunoreactivity in mucinous adenocarcinoma. (f): PAX8,40X, Nuclear inmunoreactivity in mucinous adenocarcinoma. (g): CK7, 40X, Lost inmunoreactivity in mucinous adenocarcioma.

The pathology report described the infiltration of the left ovary by a well-differentiated mucinous adenocarcinoma originating from a mature cystic teratoma. Immunohistochemistry revealed positive staining for CDX 2, CK20, SATB2 and focal PAX8, while CK7 was negative (Figure 2). The infiltration extended throughout the entire ovarian capsule, with evident capsule rupture, and also affected the external surface. However, vascular permeation was absent, and the uterine tube remained free. Deciduous changes were noted in the endometrium, and the myometrium harbored an intramural leiomyoma. Mucin lakes affected the uterine serosa, although surgical margins were clear of malignant neoplasm. Both the ovary and uterine tube were free of malignant neoplasm, as confirmed by serial sections. Subsequent cyto-reduction revealed fibroconnective tissue with acellular mucin lakes. Additionally, a scar was observed on a skin fragment, accompanied by chronic inflammatory infiltrate. Further immunohistochemistry studies (SATB2 and PAX 8) were requested to determine the primary origin. The appendicular region biopsy revealed vascularised fibroadipose tissue containing acellular mucin lakes, fibrin and necrotic tissue, with no evidence of appendicular tissue. Finally, the epiploon exhibited chronic inflammation, acellular mucin lakes and granulation tissue with foreign body giant multinucleated cells.

The patient underwent a colonoscopy, during which no lesions or tumours were found.

Subsequently, the patient received systemic chemotherapy with the FOLFOX 6 regimen (oxaliplatin, folinic acid and 5-fluorouracil every 2 weeks) based on the intestinal origin indicated by the immunohistochemistry results. She completed 12 cycles of FOLFOX 6 from 06/02/2023 to 27/07/2023.

Follow-up imaging studies revealed no abnormalities or signs of disease activity. The patient remains asymptomatic and free from malignant neoplasm. She will continue with periodic surveillance appointments.

## Discussion

The discovery of adnexal masses during pregnancy is a rare occurrence, affecting fewer than 2% of patients. Among these masses, mature cystic teratomas are the most frequent. Most of these teratomas are totally asymptomatic and are identified incidentally during ultrasound examinations, occasionally complicated by the presence of the gravid uterus [1–5].

The malignant transformation of mature ovarian cystic teratomas is a rare occurrence, happening in less than 2% of cases. Squamous cell carcinoma represents the most commonly observed malignant transformation, accounting for 75% of cases. In contrast, the transformation into mucinous intestinal adenocarcinoma is exceptionally rare, with an incidence of less than 6.8% [6, 7].

This report outlines a remarkable case of malignant transformation into mucinous intestinal adenocarcinoma within a mature ovarian cystic teratoma during pregnancy. Notably, it is the sole documentation of employing systemic chemotherapy with the FOLFOX 6 regimen to address the intestinal neoplasm, yielding highly satisfactory outcomes.

Adenocarcinomas can arise from diverse tissues, such as sebaceous glands, mammary glands, salivary glands, thyroid and epithelia of the gastrointestinal and respiratory tracts. In this instance, immunohistochemistry results demonstrated positive staining for CDX2, CK20, SATB2 and focal PAX8, alongside negative staining for CK7, suggesting a colorectal origin histology [8].

Given the rarity of such cases, there is no established standard treatment approach, requiring individualised management and resulting in variable outcomes. For young patients, like the one depicted in this case, it is advisable to commence treatment with unilateral salpingo-oophorectomy, followed by surgical staging, assessment of pathological findings and the formulation of a tailored follow-up plan [9].

Huang *et al* [10] conducted a meta-analysis report elucidating the influence of multidisciplinary cancer conferences on overall survival. The study revealed a median survival time of 30.2 months in the multidisciplinary cancer conference group, contrasting with 19 months in the control group [10].

To date, the patient has remained asymptomatic for 12 months, with no observed changes or abnormalities in diagnostic imaging. Although existing clinical practice guidelines do not specifically address the use of tumour markers for monitoring in such cases, due to the initial elevation of these markers at diagnosis, it has been decided to conduct follow-up assessments every 3–6 months during the initial 2 years, followed by annual evaluations from the third year onwards, mirroring the approach outlined in clinical practice guidelines for ovarian cancer.

## Conclusion

In summary, the malignant transformation into mucinous intestinal adenocarcinoma within a mature ovarian cystic teratoma during pregnancy is an exceedingly rare occurrence, with limited prior reports in the medical literature. Most instances of malignant transformation have been linked to the postmenopausal period. This case report stands out as one of the earliest to document the effective use of chemotherapy, specifically the FOLFOX 6 regimen, as part of the treatment protocol.

These findings underscore the importance of better understanding and awareness regarding the potential for malignant transformations in teratomas during pregnancy. These also emphasize the necessity of tailoring treatment in unique scenarios like the one depicted in this report.

Furthermore, this case underscores the significance of rigorous and prolonged follow-up for patients who have undergone this malignant transformation. The positive outcomes observed suggest that treatments such as chemotherapy with the FOLFOX 6 regimen could be a viable option in similar cases in the future. Gathering additional data and conducting research in this area is vital for enhancing our comprehension and management of this rare condition.

## Conflicts of interest

Our authors have no competing interests, and the contents of this manuscript have not been published elsewhere. There are no conflicts of interest to disclose.

## Funding

No external funding or funding by any author has been received for this study.

## Ethical statement

Studies involving human participants underwent review and approval by the Ethics Committee of Hospital Santa Rosa. The patient provided written informed consent to participate in this study.

## Authors’ contributions

The original manuscript was authored and approved by all contributing authors.

Enrique Bedoya-Ismodes: preparation of the document from its conception and design to the acquisition of the information, review of intellectual content, preparation of photos, participation in graphic designs and approval of the version sent to the editorial process.

Karem Portugal: preparation of the document from its conception and design to the acquisition of the information, review of intellectual content, data collection, preparation of tables and approval of the version sent to the editorial process.

Sandy Carmona-Lozano: bibliographic review, intellectual content and approval of the version sent to the editorial process.

Mirna Dominguez-Gonzales: preparation of photos, bibliographic review, intellectual content and approval of the version sent to the editorial process.

Jaime Torres: intellectual content, approval of the version sent to the editorial process

Nestor Juarez-Herrera: intellectual content, approval of the version sent to the editorial process.

## Figures and Tables

**Figure 1.**
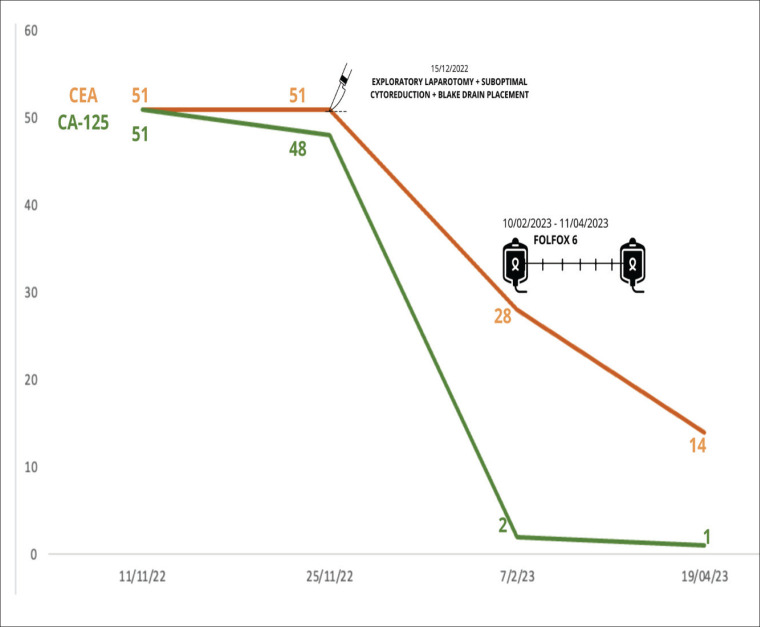
Tumour markers changes through surgery and chemotherapy with FOLFOX 6.

**Figure 1.**
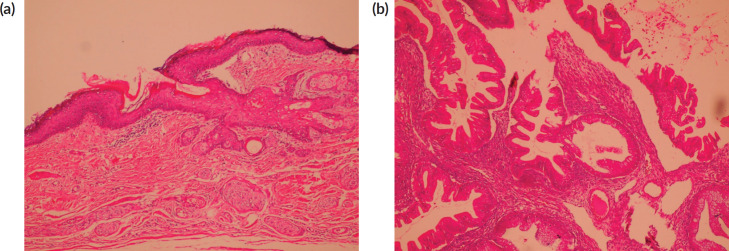
(a): Benign mature squamous epithelium (b): Malignant component displaying moderately diferentiated glandular structures (HE).
